# The St. Louis African American health-heart study: methodology for the study of cardiovascular disease and depression in young-old African Americans

**DOI:** 10.1186/1471-2261-13-66

**Published:** 2013-09-08

**Authors:** Robin R Bruchas, Lisa de las Fuentes, Robert M Carney, Joann L Reagan, Carlos Bernal-Mizrachi, Amy E Riek, Chi Charles Gu, Andrew Bierhals, Mario Schootman, Theodore K Malmstrom, Thomas E Burroughs, Phyllis K Stein, Douglas K Miller, Victor G Dávila-Román

**Affiliations:** 1 Cardiovascular Imaging and Clinical Research Core Laboratory, Cardiovascular Division, Washington University School of Medicine, 660 South Euclid Avenue, Campus Box 8086, St. Louis, MO 63110, USA; 2 Department of Psychiatry, Washington University School of Medicine, 4320 Forest Park Avenue Suite 301, St. Louis, MO 63108, USA; 3 Endocrinology, Metabolism and Lipid Research Division, Washington University School of Medicine, 660 South Euclid Avenue, St. Louis, MO 63110, USA; 4 Division of Biostatistics, Washington University School of Medicine, 660 South Euclid Avenue, Campus Box 8067, St. Louis, MO 63110, USA; 5 Mallinckrodt Institute of Radiology, Washington University School of Medicine, 660 South Euclid Avenue, St. Louis, MO 63110, USA; 6 Division of Health Behavior Research, Washington University School of Medicine, 660 south Euclid Avenue, St. Louis, MO 63110, USA; 7 Department of Neurology & Psychiatry, School of Medicine, Saint Louis University, St. Louis, MO, USA; 8 Center for Outcomes Research, Saint Louis University, 3545 Lafayette Avenue, St. Louis, MO 63104, USA; 9 Regenstrief Institute, Inc., and Indiana University Center for Aging Research, School of Medicine, Indiana University, 410 West 10th Street, Indianapolis, IN 46202, USA

**Keywords:** Coronary artery disease, Depression, Genetic analyses, African Americans, Inflammatory biomarkers, Inflammatory pathway, Serotonin-signaling pathway, Heart rate variability, Built environment, Bayesian analysis

## Abstract

**Background:**

Coronary artery disease (CAD) is a major cause of death and disability worldwide. Depression has complex bidirectional adverse associations with CAD, although the mechanisms mediating these relationships remain unclear. Compared to European Americans, African Americans (AAs) have higher rates of morbidity and mortality from CAD. Although depression is common in AAs, its role in the development and features of CAD in this group has not been well examined. This project hypothesizes that the relationships between depression and CAD can be explained by common physiological pathways and gene-environment interactions. Thus, the primary aims of this ongoing project are to: a) determine the prevalence of CAD and depression phenotypes in a population-based sample of community-dwelling older AAs; b) examine the relationships between CAD and depression phenotypes in this population; and c) evaluate genetic variants from serotoninP and inflammatory pathways to discover potential gene-depression interactions that contribute significantly to the presence of CAD in AAs.

**Methods/design:**

The St. Louis African American Health (AAH) cohort is a population-based panel study of community-dwelling AAs born in 1936–1950 (inclusive) who have been followed from 2000/2001 through 2010. The AAH-Heart study group is a subset of AAH participants recruited in 2009–11 to examine the inter-relationships between depression and CAD in this population. State-of-the-art CAD phenotyping is based on cardiovascular characterizations (coronary artery calcium, carotid intima-media thickness, cardiac structure and function, and autonomic function). Depression phenotyping is based on standardized questionnaires and detailed interviews. Single nucleotide polymorphisms of selected genes in inflammatory and serotonin-signaling pathways are being examined to provide information for investigating potential gene-depression interactions as modifiers of CAD traits. Information from the parent AAH study is being used to provide population-based prevalence estimates. Inflammatory and other biomarkers provide information about potential pathways.

**Discussion:**

This population-based investigation will provide valuable information on the prevalence of both depression and CAD phenotypes in this population. The study will examine interactions between depression and genetic variants as modulators of CAD, with the intent of detecting mechanistic pathways linking these diseases to identify potential therapeutic targets. Analytic results will be reported as they become available.

## Background

Coronary artery disease (CAD) is the leading cause of cardiovascular morbidity and mortality and one of the most prevalent chronic diseases in the industrialized world and in most developing countries [1]. Over 82 million Americans have at least one type of cardiovascular disease [2]. In 2008, CAD was responsible for one out of every six deaths in the Unites States, and each year 1.26 million Americans suffer a new or recurrent myocardial infarction [2]. Cardiac events are more prevalent in depressed patients with CAD, and patients with CAD have increased burden of depressive conditions.

Depression is a risk factor for incident CAD and for cardiac-related morbidity and mortality [3-17]. Depression is also highly prevalent in patients with CAD, At any time point, about 20% of patients with CAD are experiencing an episode of major depression, and a comparable proportion have minor depression; [18-23]. Studies have found that a history of major depression, [3,4] depression symptoms, [5-12] clinical depression, [4,13-16] and an increase in depression symptoms over time [16,17] predict incidence of heart disease and death from cardiac causes. Depression is not only a risk factor for incident CAD, but also for cardiac mortality and morbidity in patients who have established heart disease. Depression doubles the risk for cardiac events, including cardiac mortality, in the 12 months following initial diagnostic coronary catheterization and angiography [7,24]. Depression is also is a risk factor for mortality and cardiac events in patients undergoing coronary artery bypass graft surgery, [7,25,26] and following an acute coronary syndrome [27-41].

The INTERHEART study, comprised of 15,542 cases and 14,820 controls from 52 countries representing diverse world populations, identified 9 modifiable risk factors (smoking, diabetes, hypertension, abdominal obesity, fruits/vegetable intake, exercise, alcohol consumption, ApoB/ApoA1 ratio, and a psychosocial index) that account for 90% of the population attributable risk for myocardial infarction. The psychosocial index (a composite score reflective of depression, locus of control, perceived stress, and life events) was responsible for 32.5% of the population attributable risk for CAD, which was greater than the risk associated with each of hypertension, diabetes, abdominal obesity, fruit/vegetable intake, and alcohol consumption [42,43]. While depression and CAD may be two common independent disorders, it is possible that common pathophysiological mechanisms underpin both diseases.

However, the precise molecular and genetic mechanisms that may underlie both diseases remain largely unknown. Plausible pathways through which depression may increase the risk of cardiac morbidity and mortality include interactions between genetic variants and depressive phenotype (representing the environment) [44-46]. There is extensive evidence that mood disorders are heritable [47,48]. For example, heritability has been estimated on the order of 40% for unipolar depression [49]. The strongest familial risk is found in patients with recurrent major depression [50,51]. There is also evidence that cardiac risk factors and ischemic heart disease are heritable [52-55]. Recent studies have considered whether shared genetic factors help explain the relationship between depression and CAD. A variance component analysis of 2,731 male-male twin pairs from the Vietnam Era Twin Registry (mean age 42 ± 3 years) showed that shared genetic risk factors may underlie this relationship, as significant genetic correlations between depression and hypertension (r = .19) and between depression and CAD (r = .42) were found [44]. Importantly, statistical models have shown that CAD shares at least as much genetic variance with depression as it does with hypertension [56].

Inflammatory and serotonin-signaling pathways have been identified as risk factors for both CAD and depression. There is ample evidence to support a critical role for proinflammatory cytokines in the development of CAD, and inflammatory biomarkers have been associated with a variety of CAD-related traits [57,58]. The most studied of these biomarkers is the acute phase reactant C-reactive protein (CRP), which has been shown to be independently associated with the presence of carotid plaque [59] and increased carotid intima-media thickness; [60] it is also predictive of future CAD events [61-66]. Increases in inflammatory biomarkers have also been associated with depression, [67,68] and inflammation has been postulated to mediate the association between depression and CAD [69]. Serotonin, which is secreted by activated platelets, promotes thrombogenesis, mitogenesis, and proliferation of smooth muscle cells and has been shown to be associated with CAD and occurrence of cardiac events, even after adjustment for conventional risk factors [70]. Furthermore, diminished heart rate variability, a biomarker representing disordered autonomic function associated with cardiovascular mortality, has been identified in depression and anxiety. Treatment with serotonin-reuptake inhibitors may normalize heart rate variability in these patients [71]. Since inflammation and serotonin signaling pathways have been identified as risk factors for CAD events and associated with depression, it has been postulated that genetic variants in these pathways may mediate the association between depression and CAD [72].

African Americans (AAs) have traditionally been an understudied group in all aspects of biomedical and behavioral research; this is particularly true with depression, and more so in studies investigating the genetic underpinnings of depression. Nationally, AAs have higher rates of morbidity and mortality from CAD than European Americans; [73] notably, rates of death from heart disease are 1.2 times higher among AAs in the City of St. Louis compared with the national average [74]. Limited data suggest that the prevalence of major depressive disorder is at least as high in AAs as in European Americans [75,76]. Despite its importance, we are unaware of any studies examining shared biological pathways and genetic explanations for the depression-CAD relationship in AAs.

The St. Louis African American Health (AAH) cohort is a population-based cohort study of community-dwelling AAs born in 1936–1950 (inclusive). The AAH-Heart study group is a subset of AAH participants who were recruited in 2009–11 to examine the inter-relationships between depression and CAD in this population and examine potential biological pathways and genetic underpinnings that could explain the identified relationships. This publication describes the structure and content of the project. Analytic results will be reported as they become available.

## Methods/design

### Objectives

The primary objectives of AAH-Heart are three-fold: First, to identify the prevalence of CAD and depression in a population-based sample of community-dwelling AAs aged 59–75 years-old. Second, to examine the relationships between CAD and depression. Third, to evaluate interactions between depression and variants in serotonin-signaling and inflammatory pathway genes (representing gene-environment interactions) potentially to identify underlying physiological mechanisms that help explain the identified relationships between depression and CAD. Phenotypes for both CAD and depression will be defined using clinically relevant definitions based on state-of-the-art methods for characterizing these phenotypes. We will also investigate multiple biomarkers as potential mediators of previously identified depression-CAD-gene relationships.

### Study design

#### Study population

Study participants were recruited from the parent African American Health (AAH) project, a well-characterized cohort of 998 community-dwelling AAs representing the AA population in two diverse geographic and socioeconomic areas of St. Louis, MO. Participants were self-declared African American or black, born between 1936 and 1950 (inclusive; aged 49–65 at their baseline evaluation in 2000–2001), with an initial recruitment rate of 76%. They were followed through 2010 (9 years), and wave-to-wave retention was 95% among surviving participants. AAH recruitment and sampling procedures have been previously described [77]. The AAH cohort underwent 7 waves of assessments (3 in-home and 4 by telephone) that included characterization of important life course factors (e.g., reported weight; self-reported physical activity; physical, cognitive, and psychosocial functioning; comorbid diseases; socioeconomic status; and health-related quality of life). Importantly, sampling weights supplied by the contract survey organization combined with propensity score re-weighting adjustments for differential involvement in AAH-Heart permit the AAH-Heart results to represent population-based estimates of the noninstitutionalized AA population in the original parent study areas as of the 2000 Census [78].

#### Enrollment

**Figure 1:**
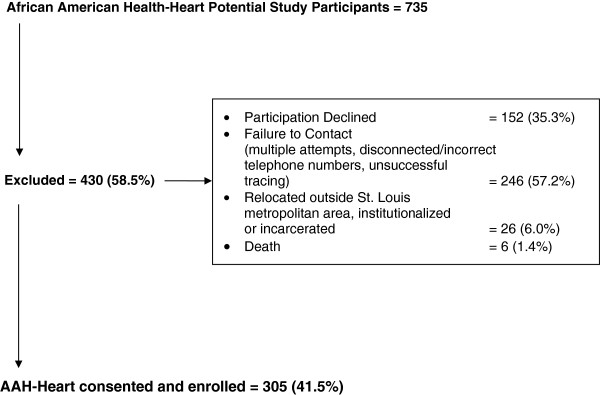
Flow chart for enrollment into the African American Health-Heart (AAH-Heart) study.

From 2009–2011, 735 AAH participants, representing the remaining AAH members available in 2009, were contacted via mail and/or telephone and asked to participate in the AAH-Heart study. Of these, 430 (58.1%) were not enrolled to the AAH-Heart ancillary study for the following reasons: declined participation (n = 152), failure to contact (e.g., multiple attempts, disconnected or wrong telephone numbers; n = 246), relocation outside the St. Louis metropolitan area, institutionalized, or incarcerated (n = 26), or death since 2009 (6). A total of 305 (41.5%) of the potential 735 AAH participants were enrolled in the AAH-Heart study (Figure 1).

### Ethical approval

The study is a joint effort of three academic institutions. The protocol was approved in full by the ethics committee at Washington University School of Medicine, and parts related to involvement of their institution were approved by the ethics committees of Saint Louis and Indiana Universities. Written informed consent was obtained from all AAH-Heart participants.

### Data collection

Data for AAH-Heart were collected from June 2009 to November 2011. All tests, questionnaires, and interviews were administered by trained personnel using standardized methods and interpreted by staff directly supervised by the investigators. Table 1 provides an overview of the variables and measurement details.

**Table 1:** Variables and instruments

**Variables**	**Instruments**
**Cardiovascular**	
Coronary, carotid, and thoracic aorta calcium scores	64-slice dual-source MDCT scanner
CIMT and carotid plaque burden	Bilateral carotid artery ultrasound imaging (mm)
ECG-derived - time domain analysis, frequency domain analysis, heart rate turbulence	24-hour Holter monitor
LV structure, LV systolic/diastolic function, valve function	Echocardiography
Heart rate	Echocardiogram
Aortic compliance- pulse wave analysis, pulse wave velocity	SphygmoCor (AtCor Medical, Sydney, Australia)
Cardiovascular history	Charleston comorbidity Index (17 items)
Blood pressure	3 times right and left arms
Ankle brachial index	Pocket fetal doppler Sonoline B
	(Clinical Guard, Atlanta, GA)
6-minute walk test	Marked distance totaled
**Emotional status**	
DSM IV diagnosis: current and lifetime major unipolar depression, generalized anxiety and panic disorder	DISH (38 items)
Hamilton rating scale: severity of current depression	
Psychiatric history	
Screen for all exclusionary psychiatric conditions	
Stressful/traumatic life events	LEC (1–15 events)
Depression	CES-D (11 items;≥9 points indicates clinically-relevant number of depressive symptoms)
**Current clinical covariates**	
Fruit and vegetable consumption	CDC questionnaire (6 items)
Medication use	Self-report
Tobacco and alcohol use	Self-report
Anthropometrics- height, weight, waist/hip	Trained research members
Physical activity	IPAQ-short (9 items)
**Laboratory &biomarker assays**	
BMP, fasting glucose, fasting lipid profile, and HgbA_1C_	Fasting blood draw
CRP, TNF-α, TNFR1, TNFR2, IL-6, TGF-β, VCAM, ICAM, MMP-9	Panomics multiplex immunoassay
	(Affymetrix, Santa Clara, CA)
**Built environment**	
Neighborhood of residence location	Questionnaire re: location; geographic information system analyses and in-person assessments as part of parent AAH study (2010)

*Abbreviations*: *BMP* basic metabolic panel, *CDC* centers for disease control and prevention, *CES-D* center for epidemiologic studies depression scale, *CIMT* carotid intima-media thickness, CRP c-reactive protein, *DISH* depression interview and structured hamilton, *DSM IV* diagnostic and statistical manual of mental disorders fourth edition, *EKG* electrocardiogram, *HbgA*_*1C*_ hemoglobin A_1C_, *ICAM* intercellular adhesion molecule, *IL-6* Interleukin-6, *IPAQ-Short* international physical activity questionnaires-short form, *LEC* life events checklist, *LV* left ventricle, *MDCT* multi-detector computerized tomography, *MMP-9* matrix metalloproteinase-9, *TGF-β* transforming growth factor-beta, *TNF-α*, tumor necrosis factor-alpha, *TNFR1* tumor necrosis factor receptor 1, *TNFR2* denotes tumor necrosis factor receptor 2, *VCAM* vascular cell adhesion molecule.

#### Cardiovascular risks and characteristics

A *Medical History*. Participants’ general health status was characterized using the Charlton Comorbidity Index [79,80]. This instrument collects data regarding cardiovascular, neurologic/cerebrovascular, connective tissue, gastrointestinal, malignant, and pulmonary diseases. Data on medications and other health-related supplements were also collected by review of a self-reported medication list and/or examination of medication bottles.

B *Cardiovascular health and behavior questionnaires*. Six questions were obtained to determine consumption of certain fruit and vegetables [81]. Participants were also questioned regarding current and past tobacco use. The nine-item International Physical Activity Questionnaire-Short Form was administered to characterize the duration and intensity of physical activity [82].

C *Anthropometric and hemodynamic measurements*. Participants’ weight, height, and waist/hip circumferences were measured. Three blood pressures were obtained in each arm after five minutes of rest in the seated position and averaged. The heart rate was obtained from a 12-lead electrocardiogram.

D *Ankle-Brachial Index (ABI)*. ABI was obtained by measuring the blood pressures in the brachial arteries and in the posterior tibial and dorsalis pedis arteries using a hand-held Doppler probe using standard techniques.

E *Six-minute walk distance test*. For this self-paced, submaximal exercise test, individuals were asked to walk without physical assistance as fast as they could during a six-minute timed period [83].

F *Coronary artery calcification*. Participants were examined using a 64-slice dual-source multi-detector computerized tomography (MDCT) scanner (Somatom Sensation 64, Siemens, Forchheim, Germany) for characterization of coronary, carotid, and thoracic aorta calcification. Scan parameters included 24×1.2 mm collimation, 1.5 mm slice thickness, 0.37 sec rotation time, spiral mode, 120 kVp, 80 mAs. Cardiac-pulsing imaging reduced radiation exposure and cardiac motion. The scan was acquired during a single breath-hold at suspended end-expiration (~5-15 sec duration). MDCT images were transferred to a workstation equipped for analysis. Intra- and inter-reader intraclass correlation coefficients for coronary calcium scores/volumes in our laboratory were ≥0.82 and ≥0.97, respectively.

G *Echocardiography*. M-Mode, two-dimensional, and Doppler echocardiographic measurements were performed for assessment of left ventricular (LV) structure and function. LV ejection fraction was calculated by the two-dimensional-derived method of discs. LV mass (LVM) was determined by the M-mode-derived cubed method and indexed to height^2.7^ (LVM/Ht^2.7^). The presence of left ventricular hypertrophy is defined as an LVM/Ht^2.7^ greater than two standard deviations above the mean (i.e. >51 g/m^2.7^ for men, >49.5 g/m^2.7^ for women) [84]. LV diastolic function was characterized as follows: 1) pulse-wave Doppler-derived transmitral indices recorded from the four-chamber view at the mitral valve leaflet tips, including the early diastolic (E-wave) and atrial (A-wave) velocities, E/A velocity ratio, E-wave deceleration time, and the isovolumic relaxation time; [85] 2) tissue Doppler imaging-derived early diastolic mitral annular velocity (E’) obtained at the septal and lateral mitral annulus from the apical four-chamber view, and averaged [85-88]. Measurements were reported as the average of three consecutive cardiac cycles, interpreted by an experience sonographer. The intraclass correlation coefficients for echocardiographic indices measured at our laboratory range from 0.75-0.88 for LV structure (i.e., LVM, LV end-diastolic and end-systolic volumes, left atrial diameter) and 0.88-0.96 for tissue Doppler imaging-derived indices of LV diastolic function (i.e., E’ at the septal and lateral annulus).

H *Carotid artery ultrasound for measurement of carotid intima-media thickness (CIMT)*. Bilateral carotid artery ultrasound imaging was performed using a 9-MHz linear array transducer of the extracranial carotid artery at the common carotid, approximately 1 cm proximal to the carotid bifurcation, using methods previously described [89]. Atherosclerotic plaque was defined as a focal intima-media thickness >1.5 mm or focal wall thickening that protruded into the lumen >0.5 mm or >50% of the surrounding CIMT.

I *24-hour Holter monitor*. Holter monitor recordings were analyzed using a CardioScan PC-based Holter analyzer, using standard research Holter analysis techniques to create an annotated beat-to-beat interbeat intervals file. Interbeat interval files were transferred to a Sun 450 computer for detailed heart rate variability (HRV) analysis using validated research software. Abnormal HRV, either markedly decreased or markedly erratic beat-to-beat changes in heart rate, is a marker for abnormal cardiac autonomic function [90]. HRV parameters included time domain measures, which are moment statistics of the amount of HRV on different scales and reflect the amount of autonomic modulation of heart rate. Detailed information on cardiac autonomic function at different underlying frequencies was obtained using frequency domain analysis of HRV. Non-linear HRV measures that assess the underlying pattern (totally random at one extreme and totally correlated at the other) were also calculated, as was heart rate turbulence (a measure of autonomic function derived from the heart rate response to ventricular premature beats, apparently reflecting baroreflex function [91-93]). Among predominantly white community-dwelling older adults in the Cardiovascular Health Study (CHS), both more abnormal non-linear HRV measures and abnormal heart rate turbulence have been associated with a high risk for cardiovascular mortality independent of traditional risk factors [94]. Also in CHS, depression has been associated with both more mortality and with non-linear HRV [95].

J *Characterization of vascular compliance*. Central aortic compliance was characterized by measurement of the pulse wave velocity (PWV) and augmentation index using the SphygmoCor™ system (AtCor, Sydney, Australia) equipped with a single high-fidelity applanation tonometer probe. For the PWV, pressures were recorded from the carotid then femoral arteries; the transit time was calculated by registration with a simultaneously recorded electrocardiogram, using methods previously described [96,97].

#### Depression and psychosocial history

A *Depression Interview*: A structured interview, the Depression Interview and Structured Hamilton (DISH), was used to diagnose current and lifetime major unipolar depression according to Diagnostic and Statistical Manual of Mental Disorders, Fourth Edition (DSM-IV) criteria; the presence and severity of current depression was determined from an embedded Hamilton Rating Scale for Depression, 17-item (HAM-D-17) [98]. The DISH also identifies depression subtypes (e.g., melancholic or atypical depression) and comorbid anxiety disorders, including generalized anxiety and panic disorder. Family history for psychiatric disorders was identified, as well as psychiatric conditions which are exclusions for this study (e.g., bipolar disorder, schizophrenia).

B *Depression Self Report Inventory*. The 11-item Center for Epidemiologic Studies Depression (CES-D) Scale was administered to assess the presence and severity of depressive symptoms. A score of ≥9 was used to define clinically significant depressive symptoms [99].

C *The Life Events Checklist* captured recent and past exposures to stressful life events [100].

#### Built environment

Each participant provided a detailed history of moves by providing the dates (month/year) and location (street number and name) of residences occupied since 2000. Additional questions probed reasons for relocations. This information will be used to determine the effects of the built environment and/or neighborhood conditions (such as nearby restaurants, grocery stores, green space, and yard and sidewalk quality) on cardiovascular and emotional health using geographic information system methods plus observational data from the parent study [78,101,102].

#### Laboratory assays

Blood was obtained by venipuncture at the time of examination. A basic metabolic profile (glucose, calcium, sodium, potassium, chloride, bicarbonate, blood urea nitrogen, and creatinine), lipid profile (low-density lipoprotein cholesterol, high-density lipoprotein cholesterol, triglyceride, and total cholesterol), and HgbA_1C_ were obtained after an overnight fast on the day of enrollment. After blood extraction and processing, serum and plasma were stored in 1 mL aliquots in O-ring tubes at -80°C pending further analysis for fasting insulin, leptin, and inflammatory biomarkers. Inflammatory biomarkers included CRP, tumor necrosis factor-alpha (TNF-α), tumor necrosis factor-receptor 1 (TNFR1), tumor necrosis factor receptor 2 (TNFR2), interleukin-6 (IL-6), transforming growth factor-beta (TGF-β), vascular cell adhesion molecule (VCAM), intercellular adhesion molecule (ICAM), and matrix metalloproteinase-9 (MMP-9), which were assayed from serum using the Panomics multiplex immunoassay magnetic bead assay kit (Affymetrix, Santa Clara, CA).

#### Genetic studies

Whole blood was collected in EDTA-treated vacuum containers and then frozen at -80°C until DNA extraction using QIAGEN Autopure LS Large Nucleic Acid Purification Instrument (Gentra Systems, Minneapolis, MN). Extracted DNA was stored at −80 C pending genotyping (in process). Genotyping is being performed using a commercially available platform at the Washington University Genome Technology Access Center. Single nucleotide polymorphisms are being selected from among inflammatory and serotonin signaling pathway genes with the goal to maximize the information content and to test variants previously implicated in cardiovascular and/or psychiatric disorders. When referent genetic sequences and/or variant data are accessed, information from African and African-American populations will be prioritized.

### Statistical analyses

Initial data processing and screening will be carried out by established procedures for data quality control and generation of derived variables (e.g., univariate analysis of individual phenotypes and identifying outliers). Version-controlled analytic datasets will be made and distributed to all approved study investigators for downstream analyses. *Latent factor analysis* will be performed on panels of observed cardiovascular findings to extract underlying patterns of data (i.e., “*traits*”) more proximally associated with CAD [103]. The latent factors will first be extracted from panels of variables representing cardiovascular attributes, and then again from panels combining both cardiovascular and depressive characteristics. By comparing results of analyses using CAD-only versus CAD + depression latent traits, subtypes of CAD potentially affected by gene-depression interactions will be identified. Simple associations between CAD/depression traits and individual genetic variants will be tested by linear or logistic regression. A modified generalized linear model accounting for gene-gene and gene-depression interactions (using both observed characteristics and extracted latent traits) will be performed to test modifying effects of depression on CAD phenotypes. Exploratory Bayesian network analyses will be performed to obtain an overview of interactions among the many factors (genetic and non-genetic) important to CAD [104,105]. Additional secondary analyses will explore whether the association between depression and CAD are mediated through LV structure/function, [106] autonomic dysfunction (e.g., HRV), [71] inflammatory biomarkers, [69] the built environment, [78] and/or health behaviors (e.g., diet, exercise, tobacco, medication use, etc.) [107]. The overarching hypothesis is that genes related to the serotonin signaling, inflammatory processes, and/or autonomic function will help explain the relationships between the depression phenotype and CAD-related phenotypes.

## Discussion

This study was designed to evaluate the contributions from depression to manifestations of CAD through common gene-gene and/or gene-environment interactions via the primary and secondary study objectives described above. This is an important and innovative study in several respects. First, the mechanisms contributing to CAD in AAs are poorly understood. For example, although AAs experience more morbidity and mortality from CAD than do European Americans, they have *less* obstructive CAD than do European Americans [73]. Second, this study uses state-of-the-art non-invasive cardiac imaging techniques to allow exquisite characterization of coronary artery calcium, CIMT, and left ventricular structure and systolic/diastolic function to evaluate manifestations of CAD. Likewise, for the evaluation of depression phenotype, detailed, validated psychiatric interviews and questionnaires were used. Third, by focusing the analyses on continuous cardiovascular phenotypes, including both raw (e.g., coronary artery calcium volume) and extracted latent traits, the power to detect significant associations will be greater than comparable analyses of dichotomized CAD traits (e.g., history of myocardial infarction). Fourth, the proposed genetic investigations are designed to take advantage of well-established genetic analytic tools supplemented by novel analytic methods developed and validated by the investigative team [103]. Fifth, multiple biomarkers and other clinical data permit the examination of physiological pathways by which depression affects CAD outcomes.

There is compelling evidence that autonomic dysregulation is one of the mechanisms by which depression contributes to CAD [108]. Studies of depressed, medically-well psychiatric patients have reported elevated levels of plasma and urinary catecholamines (primarily norepinephrine) compared to controls [109-115]. Studies have reported higher heart rates and lower HRV in depressed patients than in non-depressed controls, [111,113-120] consistent with altered cardiac autonomic nervous system function [90]. Abnormal HRV has been described as resulting from an imbalance between the sympathetic and parasympathetic regulatory control of the heartbeat [121] and/or the lack of an integrated regulation of the heartbeat [122]. (Notably, low HRV also has predicted mortality in patients with a recent myocardial infarction [123-126] and with stable coronary disease [127] and abnormal heart rate turbulence has been found to be a strong predictor of mortality after acute myocardial infarction as well [128-130]).

Biologic factors shared by both CAD and depression suggest possible common genetic pathways, such as inflammatory and/or serotonin-signaling pathways that appear to underlie both diseases. Evidence of genetic pleiotropy does not eliminate or fully explain other potential causal relationships, including pathways by which certain gene-environment interactions may cause depression and depression may in turn accelerate the development, progression, and/or morbidity associated with CAD [131]. For example, Nakatani et al. studied the “short” (S) allele of the serotonin transporter gene-linked polymorphic region (*SLC6A4* 5-HTTLPR, minor allele frequency 20%) in 2,509 Japanese post-myocardial infarction patients [132]. Depression was significantly more common among patients with (48%) than without (35%) the S allele. The S allele predicted an increased risk of cardiac events (hazards ratio = 1.69), but its effect was reduced to non-significance by adjustment for depressive symptoms, suggesting that depression may mediate the effect of this polymorphism on cardiac outcomes.

Elevated inflammatory biomarkers, particularly CRP, IL-6, and TNF-α, also have been found in medically healthy adults with depression, and in depressed patients with CAD [133-139]. These inflammatory molecules are risk markers for cardiac morbidity and mortality [57,58,63,65,66]. Thus, markers of proinflammatory and serotonin signaling pathways that have been identified as risk factors for CAD and cardiac events have also been found to be elevated in depressed patients with CAD. For this reason, genes involved in inflammatory and serotonin-signaling pathways have recently been identified as candidates for elucidating the mechanisms causing the association between depression and CAD [72].

### Limitations

Although the parent AAH project is a longitudinal study, the AAH-Heart ancillary study is cross-sectional, and thus causality cannot be determined. Second, although we can estimate effects at the population level via the original sample weights plus re-weighting adjustments based on propensity scores for inclusion in AAH-Heart, this approach is somewhat less precise than estimates based on a freshly-drawn population-based sample, albeit a great deal less expensive than the latter method. Third, although we examine a large number of biomarkers and clinical data, it is impossible to include every potential mediating, moderating, and/or confounding factor. Fourth, power predictably will be adequate for many of the planned analysis but marginal or low for others, depending on numerous issues involved in the specific analytic approach being pursued.

### Summary

AAH-Heart provides an unparalleled opportunity to study CAD-depression-gene relationships in a particularly vulnerable population, i.e., AAs entering their senior years. The investigations are designed to elucidate important pathophysiological relationships and pathways to facilitate identification of appropriate targets for clinical interventions to modify the adverse bidirectional relationships between depression and CAD, not only in AA populations but conceivably in other populations as well.

## Abbreviations

AAs: African Americans; AAH: African American Health; AAH-Heart: African American health-heart; ABI: Ankle-brachial index; BMP: Basic metabolic panel; CIMT: Carotid intima media thickness; CES-D: Center for epidemiologic studies depression, 11-item scale; CAD: Coronary artery disease; CRP: C-reactive protein; DISH: Depression interview and structured Hamilton; DSM-IV: Diagnostic and statistical manual of mental disorders, fourth edition; ECG: Electrocardiogram; HRV: Heart rate variability; ICAM: Intercellular adhesion molecule; IL-6: Interleukin-6; LV: Left ventricular; MMP-9: Matrix metalloproteinase-9; MDCT: Multi-detector computed tomography; A-wave: Peak atrial late diastolic velocity; E-wave: Peak early-diastolic transmitral velocity; PWV: Pulse wave velocity; TGF-β: Transforming growth factor-beta; TNF-α: Tumor necrosis factor-alpha; TNFR1: Tumor necrosis factor-receptor 1; TNFR2: Tumor necrosis factor receptor 2; VCAM: Vascular cell adhesion molecule.

## Competing interests

The authors declare that they have no competing interests related to this work.

## Authors’ contributors

LdlF, RMC, CCG, MS, PS, DKM, and VGD-R participated in the study conception, design, and grant writing; acquisition, analysis and interpretation of data. RRB, LdlF, JLR, TKM, and TEB participated in participant recruitment, data management, and data analysis/interpretation. MS participated in data acquisition, management, analysis, and interpretation of built environment measures. AB participated in data analysis/interpretation of MDCT images. CB-M and AER participated in analysis and interpretation of the biomarker data. All authors participated in the writing of the manuscript and provided important intellectual content. In addition, all authors read and approved the final manuscript.

## Pre-publication history

The pre-publication history for this paper can be accessed here:

http://www.biomedcentral.com/1471-2261/13/66/prepub
